# Hydrogen/deuterium exchange-mass spectrometry of integral membrane proteins in native-like environments: current scenario and the way forward

**DOI:** 10.1042/EBC20220173

**Published:** 2023-03-29

**Authors:** Waqas Javed, Damon Griffiths, Argyris Politis

**Affiliations:** 1 Faculty of Biology, Medicine and Health, School of Biological Sciences, Division of Molecular & Cellular Function, The University of Manchester, Manchester M13 9PT, U.K.; 2 Manchester Institute of Biotechnology, University of Manchester, Princess Street, Manchester M1 7DN, U.K.

**Keywords:** hydrogen-deuterium exchange mass spectrometry, liposomes, membrane proteins, membrane vesicles, nanodiscs

## Abstract

Integral membrane proteins (IMPs) perform a range of diverse functions and their dysfunction underlies numerous pathological conditions. Consequently, IMPs constitute most drug targets, and the elucidation of their mechanism of action has become an intense field of research. Historically, IMP studies have relied on their extraction from membranes using detergents, which have the potential to perturbate their structure and dynamics. To circumnavigate this issue, an array of membrane mimetics has been developed that aim to reconstitute IMPs into native-like lipid environments that more accurately represent the biological membrane. Hydrogen/deuterium exchange-mass spectrometry (HDX-MS) has emerged as a versatile tool for probing protein dynamics in solution. The continued development of HDX-MS methodology has allowed practitioners to investigate IMPs using increasingly native-like membrane mimetics, and even pushing the study of IMPs into the *in vivo* cellular environment. Consequently, HDX-MS has come of age and is playing an ever-increasingly important role in the IMP structural biologist toolkit. In the present mini-review, we discuss the evolution of membrane mimetics in the HDX-MS context, focusing on seminal publications and recent innovations that have led to this point. We also discuss state-of-the-art methodological and instrumental advancements that are likely to play a significant role in the generation of high-quality HDX-MS data of IMPs in the future.

## Introduction

Integral membrane proteins (IMPs) constitute an important therapeutic target, due to their involvement in a range of biological functions including signal transduction, transport of metabolites, and efflux of cytotoxic compounds [1,2]. When in their native environment, IMPs are embedded into a heterogeneous mixture of lipids that modulate their structure, dynamics, and function [3]. Historically, the characterisation of IMPs has relied on their extraction from membranes into a wide selection of detergents that act as surrogate for their native lipid environment [4]. Whilst detergents can solubilise IMPs in buffers amenable to structural and biochemical analyses, possible perturbation of IMP structure and dynamics remains a concern [5,6]. Consequently, great effort has been spent on developing an array of different membrane mimetics that more accurately represent the native lipid environment [7].

To better understand IMP structure–function relationships, information on protein dynamics is required in addition to high-resolution structures [8]. Hydrogen/deuterium exchange-mass spectrometry (HDX-MS) has emerged as a powerful tool for probing protein dynamics [9]. HDX-MS measures mass increases associated with the isotopic exchange between amide hydrogens of the polypeptide backbone and deuterium in the surrounding solvent; a reaction commonly termed HDX. In folded proteins, the rate of HDX is determined by protein higher-order structure; namely, amide hydrogen solvent accessibility and H-bond stability [10]. Thus, HDX-MS readouts can report on changes in protein dynamics and localise them to specific structural regions. The continued evolution of HDX-MS methodology has allowed us to begin coping with increasingly native-like mimetic environments. This has, in turn, resulted in an increasing number of publications utilising HDX-MS to probe the dynamics of mimetic reconstituted IMPs, thereby providing greater insight into their lipid-modulated mechanism of action and regulation [11].

The emphasis of the present mini-review is on the current strategies used, and innovations that led to, the amenability of membrane mimetics in HDX-MS analyses. Technical aspects of HDX-MS have been reviewed elsewhere [9,12]. In the ‘current scenario’ section, we compile all available results from publications utilising HDX-MS for the analyses of IMPs reconstituted into lipid-containing mimetics, as well as pioneering work involving *in vivo* HDX-MS. Furthermore, we discuss the distinct advantages and disadvantages of each, as well as strategies for maximising peptide output. In the ‘way forward’ section, we discuss state-of-the-art methodological and instrumental innovations being developed to improve analyses, with particular focus on automation of delipidation processes and increasing liquid chromatography-mass spectrometry (LC-MS) peak capacity via subzero chromatography and orthogonal ion mobility (IM) separations.

## Current scenario

### Membrane mimetics

**Figure 1:**
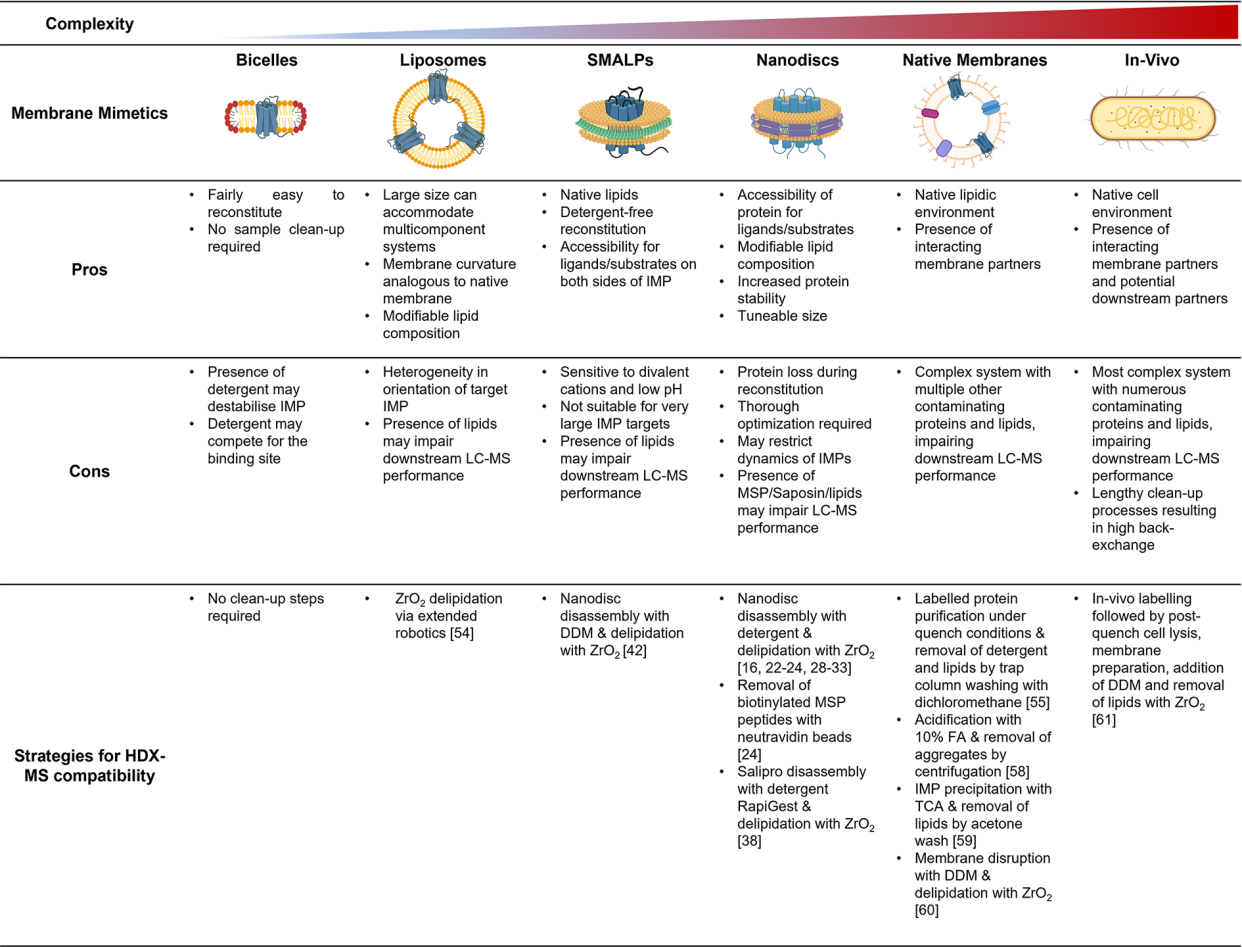
Summary of the pros, cons, and strategies for HDX compatibility of IMPs reconstituted into lipid containing mimetics of increasing complexity Figure was prepared using BioRender.

The membrane environment is crucial for optimal functioning of IMPs. Membrane curvature, fluidity, and lipid composition play important roles [3]. In fact, protein–lipid interactions have been shown to directly impact the function and stability of IMPs [13–16]. Several lipid-containing membrane mimetics have been developed over the years, each having some benefits over the others [5,7]. These have been summarised in Figure 1 along with their pros, cons, and strategies for HDX compatibility.

### Nanodiscs

Nanodiscs are self-assembled phospholipid bilayers held together by membrane scaffold protein (MSP), developed by the Sligar lab [17,18]. MSP is derived from the human serum apolipoprotein A-1. A range of MSP constructs are available to generate nanodiscs of variable diameters accommodating IMP of various sizes [18]. The self-assembly of nanodiscs occurs when detergent is removed from detergent-solubilised IMP in the presence of phospholipid/detergent micelles and MSP [17,19]. In the context of HDX-MS, nanodiscs provide an invaluable tool for the study of IMP in native-like lipid environment [20,21]; the protein being accessible for ligands/substrates from both sides. However, the contaminating peptides from MSP increase MS spectral complexity and the additional sample clean-up steps often complicate HDX-MS workflows. The study of nanodisc-incorporated IMP by HDX-MS has been reviewed elsewhere, so this section will mainly focus on the strategies used to make nanodiscs HDX-compatible and innovations that led to increased peptide output [20,21]. Nanodisc-reconstituted IMPs studied by HDX-MS have been summarised in Table 1.

**Table 1:** Summary of nanodisc-reconstituted IMP studied by HDX-MS

IMP	Family	Study	Purification detergent	MSP	Lipids	Ratio of IMP:MSP: lipids	Reconstituted nanodisc purification method	Nanodisc disassembly (final conc.)	Sequence coverage (%)	Ref.
**GGCX**	Carboxylase	Proof of concept	0.5% CHAPS 0.2% DOPC	1D1	DOPC	1:20 IMP:MSP	SEC	Na-cholate:lipids 25:1	45	[22]
**GGCX**	Carboxylase	IMP-Ligand Interaction	0.5% CHAPS 0.2% DOPC	1D1	DOPC	1:20:1200	SEC	Na-cholate:lipids 25:1	42	[23]
**LeuT**	NSS	Conformational changes	0.05% DDM	1E3D1	POPC:POPG 3:2	1:20:1000	IMAC SEC	0.006% DDM	65	[24]
**LeuT**	NSS	Conformational changes	0.05% DDM	1D1	POPC:POPG 3:2	1:10:800	IMAC SEC	Na-cholate:lipids 25:1	30	[28]
**XylE LacY**	MFS	Effect of lipid composition on conformational dynamics	0.02% DDM	1E3D1	POPE/POPC: POPG:CL 7:2:1	1:8:480	IMAC SEC	0.1% DDM	85	[16]
**P-gp**	ABC Transporter	Conformational changes	0.1% DDM	1D1	DMPC	1:10:800	SEC-HPLC	Na-cholate:lipids 25:1	38	[30]
**P-gp**	ABC Transporter	Effect of cholesterol on conformational dynamics	0.1% DDM	1D1	DMPC/ Cholesterol	1:10:70	IMAC SEC	0.1% DDM	52	[31]
**P-gp**	ABC Transporter	Effect of substrate/inhibitor on conformational dynamics	0.1% DDM	1D1	DMPC	1:10:70	IMAC SEC	0.1% DDM	50	[32]
**BmrA**	ABC Transporter	Drug release mechanism	0.035% DDM 0.03% Na-cholate	1E3D1	*E. coli* total lipid extract	1:5:400	IMAC	0.035% DDM 0.03% Na-cholate	93	[33]

Abbreviations: ABC, ATP-binding cassette; BmrA, Bacillus multidrug-resistance ATP; CHAPS, 3-((3-cholamidopropyl) dimethylammonio)-1-propanesulphonate; DDM, detergent *n*-dodecyl β-D-maltoside; DMPC, 1,2-dimyristoyl-*sn*-glycero-3-phosphocholine; DOPC, 1,2-dioleoyl-*sn*-glycero-3-phosphocholine; GGCX, gamma-glutamyl carboxylase; IMAC, immobilised metal ion affinity chromatography; MFS, major facilitator superfamily; NSS, neurotransmitter:sodium symporter; P-gp, P-glycoprotein; POPC, 1-palmitoyl-2-oleoyl-*sn*-glycero-3-phosphocholine; POPG, 1-hexadecanoyl-2-(9Z-octadecenoyl)-*sn*-glycero-3-phosphoglycerol; SEC, size exclusion chromatography.

The seminal study integrating HDX-MS and nanodiscs for IMP was undertaken by Engen’s group [22]. This study led to 45% sequence coverage of the human gamma-glutamyl carboxylase (GGCX) in 1,2-dioleoyl-*sn*-glycero-3-phosphocholine (DOPC) nanodiscs. The key steps in the HDX workflow included: (i) Disassembly of nanodiscs by the addition of cholate at the quench step, which increased peptide recovery. (ii) Removal of phospholipids by using zirconium dioxide beads (ZrO_2_). At acidic conditions, ZrO_2_ acts as a Lewis acid with net positive charge having affinity towards polyoxy anions with high binding constant for phosphate. As such, ZrO_2_ can be used to bind to phospholipids after nanodisc disassembly and then be subsequently removed via filtration.

Conformational changes were tracked in nanodisc-embedded ligand-bound vs unbound GGCX by Parker et al. [23]. GGCX is involved in post-translational modifications of Vitamin K-dependant (VKD) proteins implicated in blood coagulation. Although the sequence coverage was a mere 42%, useful information was gained in the context of structural rearrangement of GGCX upon binding to its ligand ‘propeptide’. Propeptide is an 18 amino acid sequence on VKD proteins required for GGCX-binding. Propeptide binding induced structural stability in GGCX with significant protection observed at the propeptide-binding region and the catalytic glutamate-binding site leading to a catalytically stable conformation.

Adhikary et al. focused on the model bacterial neurotransmitter:sodium symporter (NSS) leucine transporter (LeuT) reconstituted in 1-palmitoyl-2-oleoyl-*sn*-glycero-3-phosphocholine (POPC) and 1-hexadecanoyl-2-(9Z-octadecenoyl)-*sn*-glycero-3-phosphoglycerol (POPG) nanodiscs [24]. Their mammalian counterparts are involved in the sodium-dependant reuptake of neurotransmitters in the synaptic cleft making them an important target for psychotropic drugs. They are known to function via an alternating access mechanism, with the substrate-binding site alternatively accessible to either side of the membrane via conformational changes [25,26]. A combination of HDX-MS and molecular dynamics (MD) simulations was used to trace signature profiles of the outward facing (OF) and inward facing (IF) conformations using the WT and Y268A mutant, respectively. The WT LeuT in the presence of excess sodium shifts the equilibrium towards the OF conformational ensemble, whereas the Y268A mutant mainly adopts the IF conformations [27]. The results highlighted the role of transmembrane helix (TM) 1a, TM7, extra cellular loop (ECL) 2 and ECL4 in the conformational transition of LeuT during its transport cycle. To maximise sequence coverage, MSP peptides were removed using neutravidin ultraLink beads at the quench step, thereby decreasing MS spectral complexity and allowing better identification and monitoring of target peptides.

Merkle et al. investigated the substrate translocation mechanism of LeuT in detergent *n*-dodecyl β-D-maltoside (DDM) by varying substrate/ion composition (leucine, Na^+^, K^+^, and Cs^+^), aiming to shift the equilibrium towards distinct conformational ensembles, representative of different stages of the transport cycle. An interesting observation was made regarding peptides in the intracellular region showing EX1 HDX kinetics. Consequently, a control experiment in native-like environment was undertaken by reconstituting LeuT into nanodiscs, which also confirmed this feature. The group proposed a detailed transport cycle of LeuT, highlighting that the partial unwinding of specific TM regions trigger the switch from substrate-bound OF occluded to IF conformation [28].

**Figure 2:**
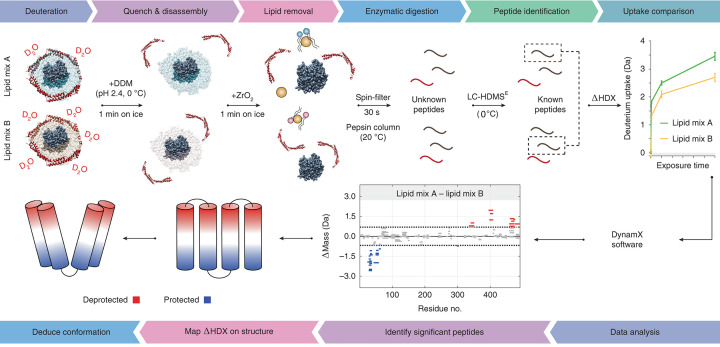
Workflow for identification of lipids modulating nanodisc reconstituted IMP conformational dynamics Figure modified from Martens et al. [29]. IMPs are reconstituted into nanodiscs with differing lipid composition and labelled with D_2_O over several different timepoints. The reaction is then quenched by decreasing pH and temperature to 2.4 and 0°C, respectively, followed by nanodisc disassembly by addition of DDM detergent. Lipids are then removed by off-line addition of zirconia beads and proteins digested using an on-line pepsin column at 20°C. The resulting peptides are then identified and monitored via LC-MS. Peptide deuterium uptake is then quantified and compared between states using DynamX, followed by peptide-level statistical significance testing using Deuteros [75]. Differential deuterium uptake can be mapped onto high-resolution structures for data interpretation.

The major facilitator superfamily (MFS) is known to be the largest among the secondary active transporters. Like LeuT and P-glycoprotein (P-gp), they function via an alternating access mechanism, but the role of lipids in the conformational landscape is poorly understood. Martens et al*.* showed that the native *E. coli* phosphatidylethanolamine (PE) lipids shifted the conformational equilibrium of the two MFS transporters D-xylose-proton symporter (XylE) and lactose permease (LacY) to the IF conformation relative to the non-native phosphatidylcholine (PC) lipids [16]. The group used concentrated nanodisc samples coupled with IM and high-pressure on-line digestion (7000 psi) to achieve an impressive 85% sequence coverage. This work led to a more detailed protocol to study lipid-modulated conformational changes in IMP using HDX-MS and MD simulations (Figure 2) [29].

The group of William Atkins performed extensive HDX-MS studies on nanodisc-reconstituted P-gp [30–32], an ATP-binding cassette (ABC) transporter involved in the efflux of xenobiotics. In their initial study, they compared the conformational dynamics of P-gp in the ligand-free state vs the ADP-trapped state induced by vanadate. This was done in DDM/lipid micelles and after reconstitution in 1,2-dimyristoyl-*sn*-glycero-3-phosphocholine (DMPC) nanodiscs [30]. Although the sequence coverage was just ∼40%, it was sufficient to observe an overall modest protection in the ADP-trapped state. In nanodiscs, deuterium uptake was comparatively less, suggesting more restricted protein dynamics. Another interesting feature was the presence of peptides showing EX1 kinetics. These peptides largely belonged to conserved sequences of the nucleotide-binding domains (NBDs) and the intracellular loops (ICLs), known to be implicated in its conformational transitions, thereby providing evidence of a more complex conformational landscape at the resting state. This conformational heterogeneity was considered a prerequisite for the substrate promiscuity of P-gp for conformational selection of allocrites of varying size range.

Clouser et al. compared the effect of cholesterol on P-gp reconstituted in DMPC/cholesterol nanodiscs vs DMPC nanodiscs [31]. A sixfold stimulation of ATPase activity was observed in the presence of cholesterol. HDX-MS revealed that cholesterol induced allosteric asymmetric changes in the NBDs, potentially mediated through intracellular helices (ICHs). Deprotection was observed in NBD1 and the adjacent ICH4, whereas protection was observed in NBD2 and the neighbouring ICH3. Interestingly, most of these peptides showed bimodal distributions, a hallmark of EX1 kinetics. Deconvolution of some bimodal peptides led to the conclusion that cholesterol shifts the conformational equilibrium towards a more catalytically competent conformation.

Different drugs/substrates affect P-gp differently at the NBDs, in turn affecting ATP hydrolysis, and at the transmembrane domains (TMDs), in turn affecting allocrite translocation. Moreover, some may affect the coupling between these two domains. To understand the molecular mechanism underlying this difference, comparisons were drawn between the effect of substrate vinblastine and the transport inhibitor zosuquidar on the nucleotide free, prehydrolytic and posthydrolytic states of P-gp in DMPC nanodiscs [32]. A major difference was seen in the zosuquidar incubated posthydrolytic state where an overall deprotection was observed at the TMDs and NBDs. This provided evidence that zosuquidar alters stability of the posthydrolytic state, in turn causing catalytic cycle disruption by uncoupling ATP-hydrolysis and transport.

Chaptal et al. resolved the high-resolution structure of another ABC transporter Bacillus multidrug-resistance ATP (BmrA), a bacterial homologue of P-gp, in the substrate-bound OF conformation [33]. It was shown using HDX-MS on BmrA in detergent and after reconstitution into nanodiscs, that the plasticity of TM1 and TM2 drives drug release in the OF conformation. Another key feature was the 93% sequence coverage in nanodiscs achieved using a C4 ultra-performance liquid chromatography (UPLC) column that potentially helped in the elution of more hydrophobic peptides. In addition, DDM/cholate, in a ratio shown to significantly reduce the estimated detergent-belt size, was used to disassemble the nanodiscs at the quench step [34]. Pre-equilibrated magnetic zirconium beads were then used to remove the phospholipids.

### Salipro

Saposin A (Sap A) lipoprotein nanoparticle system is another nanodisc technology that uses Sap A peptides to surround the IMP in lipid bilayer [35,36]. The major advantage of Sap A nanodiscs is their adaptability to any IMP size, in contrast with MSP nanodiscs, where optimisation of the different sized MSP constructs is required [37].

Zhou et al. tested the feasibility of Salipro with HDX-MS and other structural MS techniques, using reconstituted Ferroportin, an MFS iron exporter [38]. The group obtained 92% sequence coverage by using the surfactant RapiGest and 4 M urea in the quench for nanodisc disassembly and protein unfolding, respectively. Phospholipids were subsequently removed using ZrO_2_.

### SMALPs

Styrene-maleic acid lipid particle (SMALP) technology uses styrene-maleic acid (SMA) polymers to solubilise IMPs directly from the membrane along with native lipids [39]. This offers a detergent-free strategy and bypasses the potential detergent-induced perturbation in IMP stability and function. However, their sensitivity to low pH and divalent cations restricts their true potential in HDX-MS applications [40]. Recently, the emergence of SMA-like copolymers, shown to be stable under these conditions, could potentially overcome these challenges [41].

Reading et al. took advantage of this technology to study the effect of different lipid compositions on the rhomboid protease GlpG from *E. coli* [42]. They modulated the native lipid composition by overexpressing the protein in two different strains of *E. coli*, BL21 and C43, and by inducing at two different temperatures. BL21 was shown to have more PE and cardiolipin (CL) compared with C43, whereas C43 induced at a lower temperature had less CL content and increased fatty acid chain unsaturation in comparison with standard C43. It was shown that the increased membrane fluidity as a result of this unsaturation led to increased HDX at the cytosolic domain, linker region, and TM1 of GlpG. This highlighted the role of these regions in GlpG function via their interaction with membrane lipids. The authors adopted Engen’s nanodisc workflow for removal of phospholipids but used DDM for SMALP dissociation at quench step, leading to 80% sequence coverage.

### Bicelles

Bicelles are membrane bilayer discs formed by mixing short-chain phospholipids or detergents with long-chain phospholipids [43,44]. The ease of reconstitution and no sample clean-up for HDX-MS studies add to their advantages. On the other hand, the presence of detergent may perturbate the target IMP structure and function.

Pulsed HDX-MS was used to study for the first time, according to the authors, the refolding kinetics of the IMP bacteriorhodopsin, a light-driven proton transporter [45]. The refolding of the SDS-denatured bacteriorhodopsin was initiated by mixing it with DMPC and 3-((3-cholamidopropyl) dimethylammonio)-1-propanesulphonate (CHAPS) bicelles [46]. A custom built four-syringe device was used to undertake pulsed HDX in offline mode and protein mass was subsequently analysed using SEC-MS. The results indicated multistep-refolding events leading to a rather slow recovery of the secondary structure over 10 s and longer timescale.

Duc et al. investigated the feasibility of bicelles for the study of G protein-coupled receptors (GPCRs) [47]. A comparison was made between DMPC and 3-((3-cholamidopropyl) dimethylammonio)-2-hydroxy-1-propanesulphonate (CHAPSO) bicelles vs the classical DDM detergent for the three model GPCRs β2-adrenergic (β2AR), μ-opioid receptor, and protease-activated receptor 1. Bicelles outperformed DDM with around 95% sequence coverage for the tested proteins and a significantly higher peptide redundancy.

Komolov et al. probed the interaction of GPCR with GPCR kinase 5 (GRK5) using β2AR reconstituted in DMPC, CHAPSO, and PIP2 bicelles [48]. Protection observed by HDX-MS at TM5 and the C-terminus of β2AR, and N-terminal lipid-binding domain and Regulator of G protein-signalling homology bundle of GRK5, provided evidence of direct interaction at these sites. It was also observed that receptor-binding induces allosteric changes at the GRK5 catalytic domain. Overall, the group comprehensively characterised the structural mechanism by which activated GPCRs get phosphorylated by GRKs, using a range of biophysical and biochemical techniques.

### Liposomes

Liposomes are lipid bilayer vesicles that have been successfully used for structural and functional analysis of IMPs [49,50]. Early HDX-MS work on liposomes containing hydrophobic peptides was a milestone in the development of electrospray ionisation-mass spectrometry (ESI-MS) application for IMPs [51–53]. Liposomes are very close to native membranes in membrane curvature, offer tuneable lipid composition, and can accommodate multicomponent systems owing to their large size. Nevertheless, the heterogeneity in orientation of reconstituted IMP may complicate interpretation and reproducibility of results.

Anderson et al. outlined an automated phospholipid removal method using FcγRIIa reconstituted in liposomes as a model system [54], which is detailed in the following section.

### Native membranes

Membrane vesicles provide a native environment with increased structural and functional stability for IMP. The presence of interacting membrane partners and lipids may strengthen the physiological relevance of data, but at the same time, can further exacerbate technical challenges.

Eric Forest’s group undertook pioneering work on HDX-MS of IMPs in their native mitochondrial membrane. They investigated the bovine ADP/ATP carrier, belonging to the mitochondrial carrier family, located in the inner mitochondrial membrane [55]. Two transport inhibitors carboxyatractyloside (CATR) and bongkrekic acid (BA) were used to lock the transporter at different stages of the cycle. The carrier, in isolated mitochondrial membranes, was first incubated with either CATR or BA, and then labelled by dilution in deuterated buffer. Once the reaction was quenched, the protein in mitochondrial membranes was solubilised in triton X-100, and then further purified using a gravity flow hydroxylapatite (hydroxylated calcium phosphate) packed column. The protein elution was achieved under pressure using a piston to limit back exchange and then digested using a pepsin column. Another important step was the removal of detergent and lipids in the trap column using dichloromethane. The peptides were separated using C18-reversed phase high-performance liquid chromatography (HPLC) column leading to 58% sequence coverage. The present study highlighted significant differences in the conformational dynamics of the carrier in membranes vs in detergent [56].

Mehmood et al. used HDX-MS to gain an insight into the transport cycle of BmrA purified in DDM. The group also followed global HDX on BmrA in inverted membrane vesicles (IMVs), either in ligand-free resting sate or ADP-trapped state stabilised by vanadate. Interestingly, BmrA showed less deuterium uptake in IMVs relative to detergent at the resting state, pointing towards a more structurally compact/stable conformations of protein in membranes. Overall, the protein, in both detergent and membranes, showed high level of deuterium uptake at the resting state and protection against exchange in the ADP-trapped state, known to shift the equilibrium towards the OF conformational ensemble. The intact protein for global exchange was separated from lipids using the C18 HPLC column [57].

The group of Lars Konermann was one of the first to undertake HDX-MS on *E. coli* IMVs overexpressed with FoF1 ATP synthase, which uses the proton motive force (PMF) to drive ATP synthesis [58]. The conformational dynamics of this motor were studied using various catalytically active or inhibited states. The authors observed destabilisation in the C-terminus of the γ-shaft in conditions where protons were pumped into the IMVs, driven by ATP hydrolysis against the PMF. Proposedly, this was due to torsional stress-mediated destabilisation of amide hydrogens of the γ polypeptide backbone, induced by its rotation at the apical bearing. The HDX-MS workflow included acidification of the labelled IMVs with 10% formic acid (FA) to quench the reaction, followed by in-solution digestion and centrifugation to remove the insoluble aggregates.

Donnarumma et al. took advantage of *E. coli* secreted outer membrane vesicles (OMVs) to study the structural arrangement of the outer membrane porin, outer membrane protein F (OmpF), naturally overproduced in these vesicles [59]. They devised an HDX-compatible protocol to remove lipids by acetone wash after trichloroacetic acid (TCA) precipitation of the labelled protein. The results confirmed the trimeric assembly of the protein in native membranes.

The group of Sosnick used BtuB, an *E. coli* TonB-dependant vitamin B12 transporter, to highlight structural changes induced by vitamin B12 binding and transport [60]. HDX-MS was performed on native *E. coli* outer membranes (OM), separated from inner membranes using sarkosyl. The important steps of the workflow included addition of DDM to the quench and removal of lipids by ZrO_2_. The major finding was the allosteric liberation of the ionic lock upon B12 binding followed by the binding of the N-terminus of BtuB to TonB in the periplasm. The authors did not find any evidence of BtuB-TonB pore formation, which they suggested might require energy input from the inner membrane missing in the study.

### *In vivo* HDX

Although the study of IMPs in native membranes is a milestone for HDX-MS, it may lack some features only present in intact cells, such as presence of potential downstream partners. Nonetheless, the lengthy sample clean-up processes result in high back exchange, and the numerous contaminating proteins and lipids can impair downstream LC-MS performance.

**Figure 3:**
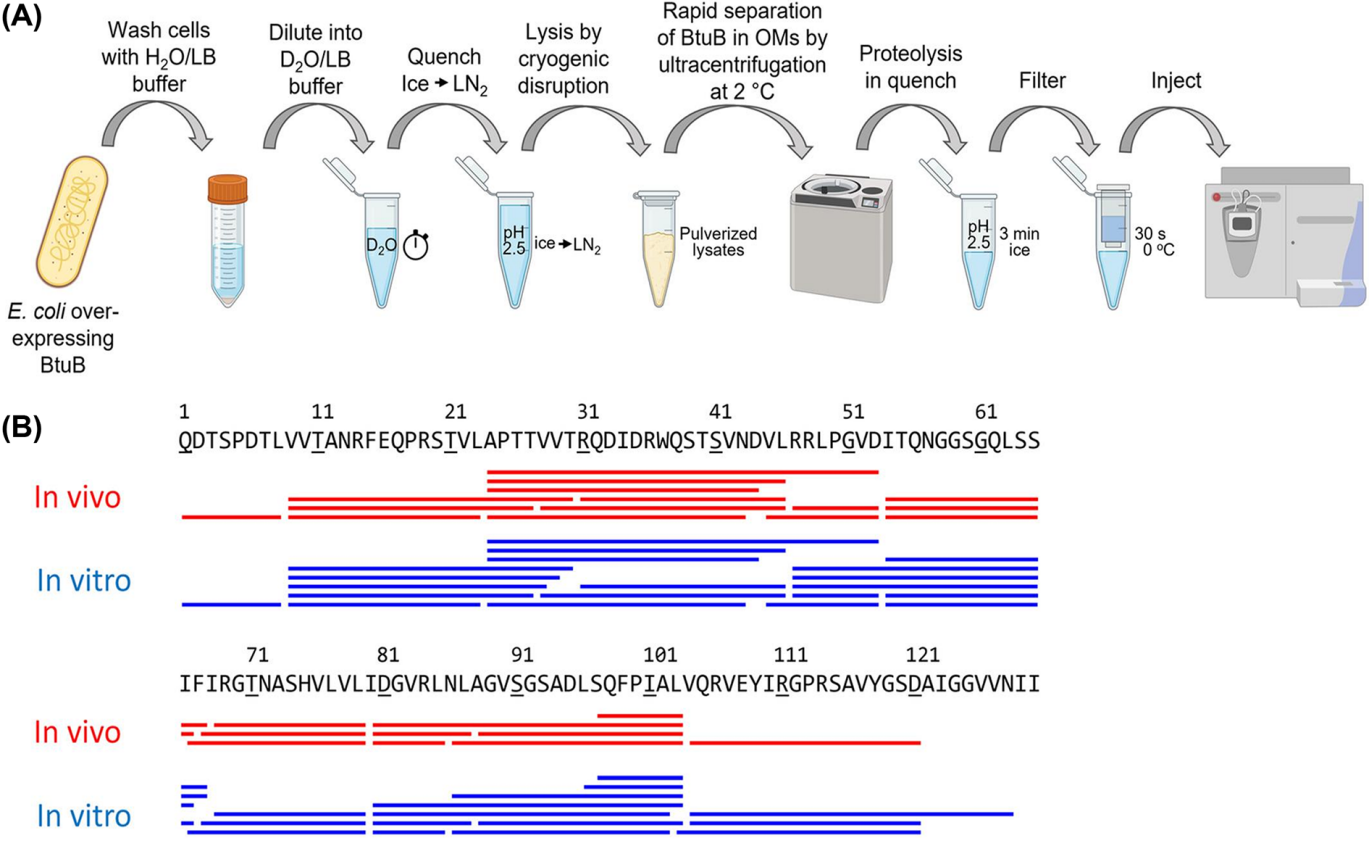
Experimental workflow and sequence coverage for *in vivo* HDX-MS on BtuB Figure taken from Lin et al. [61] with permission from the publisher (Protein Science). (**A**) For *in vivo* HDX-MS, living *E. coli* cells overexpressing the protein of interest (in this case BtuB) are diluted in D_2_O buffer supplemented with a carbon source. The reaction is then quenched by dropping buffer pH to 2.5 and flash freezing the cells with liquid nitrogen; the latter also acting to lyse the cells via cryogenic disruption. OMVs containing the proteins of interest are then isolated by ultracentrifugation at pH 2.5 and 2°C. This was followed by in solution proteolytic digestion, addition of ZrO_2_ for delipidation, and removal of peptides via centrifugal spin filtration. Peptides were then subjected to LC-MS analyses for peptide identification and monitoring. (**B**) Both *in vivo* and *in vitro* HDX-MS experiments provided high levels of BtuB sequence coverage; 93% and 98%, respectively.

The Sosnick group developed a protocol for *in vivo* HDX-MS using the model IMP BtuB (Figure 3) [61]. The protein was labelled in live *E. coli* cells by transferring them to deuterated growth media. After labelling, the reaction was quenched, and cells lysed by cryogenic pulverisation. The membranes were collected by ultracentrifugation under quench conditions. Thereafter, their OM HDX-MS protocol was followed as described above, obtaining 93% BtuB sequence coverage [60]. Overall, the *in vivo* data validated the group’s results obtained in OMVs, though a higher back exchange was observed owing to the lengthy postquench processes. From methodological perspective, the authors also obtained HDX data on various other endogenous *E. coli* proteins with reasonable sequence coverages.

## The way forward

### The need for automated delipidation

The introduction of membrane mimetics or cell lysate into HDX-MS workflows can exacerbate technical challenges. These challenges are primarily driven by the presence of large quantities of lipids, which cause analyte ionisation suppression, increase MS spectral complexity, impair chromatographic performance, and reduce proteolysis efficiency; all of which reduce target coverage and redundancy. Thus, the removal of lipid prior to LC-MS analyses is often a prerequisite for successful HDX-MS of mimetic reconstituted and *in vivo* IMPs.

HDX-MS analyses of IMPs have, thus far, relied on off-line manual delipidation procedures. However, manual lipid clean-up can increase measurement error across triplicate experiments, thereby potentially obscuring subtle differences in dynamics across different conditions. Consequently, several recent publications have sought to address this issue via automated delipidation.

### ZrO_2_ delipidation via extended robotics

Anderson et al. published the first automated delipidation method for HDX-MS in which they modified a LEAP HDX robot to perform ZrO_2_-mediated delipidation analogous to Engen’s original protocol [54]. However, rather than using centrifugal spin filtration to remove lipid-bound ZrO_2_, the robot used an X-press module that applied downward pressure on a polyethersulphone nanofilter cartridge containing the sample-ZrO_2_ mixture. This filtered protein into an upper reservoir for LC-MS analysis, whilst lipid-bound ZrO_2_ was retained for disposal. The authors successfully employed this system to perform HDX-MS on FcγRIIa in liposome, achieving 66% sequence coverage. Since this initial publication, this technology has been commercialised as Trajan’s LEAP HDX automation with lipid filtration.

### On-line phospholipid trapping

Hammerschmid et al. proposed chromatographic phospholipid trapping columns containing ZrO_2_ or titanium dioxide beads (TiO_2_) [62]. In their method, a Waters HDX manager and UPLC was modified with an additional valve and ZrO_2_/TiO_2_ column to perform on-line protein- or peptide-level delipidation. Furthermore, an additional binary solvent manager (BSM) was used to wash and re-equilibrate ZrO_2_/TiO_2_ columns simultaneously to the LC separation, meaning columns were regenerated without reducing analytical through-put.

When evaluating delipidation efficiency, Hammerschmid et al. reported a >100 to 1000-fold reduction in POPC when using ZrO_2_/TiO_2_ trap columns [62]. Furthermore, an approximately 96% delipidation efficiency was reported for *E. coli* lipid extract, demonstrating a capacity to handle complex lipid mixtures. Interestingly, addition of DDM to quench was found to reduce delipidation efficiency, potentially caused by steric hindrance from micelle formation. In contrast, Fos-choline-12 exhibited no detrimental impact, suggesting it could be a more appropriate quench additive for mimetic disassembly.

Nonspecific protein adsorption onto the ZrO_2_ column was also reported by Hammerschmid et al. [62]. Whilst attempting to reduce nonspecific binding, less severe adsorption was observed over technical replicates, suggesting ‘blocking’ of nonspecific binding sites via protein saturation. In addition to using a glycine hydrochloric acid-based quench, they proceeded to ‘block’ ZrO_2_ columns with 3% bovine serum albumin prior to sample delipidation, which was found to increase target peptide intensity four- to fivefold. This strategy could potentially be adopted more widely, even for off-line ZrO_2_ delipidation. Finally, acriflavine-resistance protein b (AcrB) nanodiscs were analysed that generated an impressive 82.7% sequence coverage, demonstrating its high applicability for HDX-MS applications.

### On-line size exclusion chromatography

To our knowledge, Calvaresi et al. were the first to utilise size exclusion chromatography (SEC) for automated on-line delipidation [63]. In short, a SEC column was installed on a Waters HDX manager under two configurations; configuration 1, where SEC was positioned between the injection loop and protease column for protein-level delipidation, and configuration 2, where SEC was positioned between the protease column and trap valve for peptide-level delipidation. In both configurations, isolated contaminants could subsequently be directed to waste to prevent detrimental effects on downstream LC-MS.

In control experiments, Calvaresi et al. reported only a 6% lower efficiency compared with off-line TCA precipitation [63]. Furthermore, initial experiments involving POPC/ubiquitin mixtures suggested that lipids were retained via strong binding interactions with the SEC stationary phase, resulting in near-complete delipidation. Thus, this method demonstrates great utility for mimetics with lower lipid quantities. SEC-lipid binding capacity was, however, reported to reduce when injecting high lipid quantities over successive injections. Despite this, using configuration 2, an impressive 73% sequence coverage was obtained for NadA in OMVs, despite constituting only 8.8% of total protein content within the OMVs.

Whilst these methods have shown great utility in HDX-MS applications, each have their own advantages and disadvantages that have been summarised in Table 2.

**Table 2:** Comparative advantages and disadvantages of automated delipidation methods for HDX-MS

Method	Advantages	Disadvantages
LEAP HDX robot lipid filtration [54]	• Robot handling steps can be fine tuned • Uses established ZrO_2_/TiO_2_ protocols shown to work well with complex membrane mimetics • No need for ZrO_2_ or TiO_2_ regeneration	• Requires purchase of robotics • Only protein-level delipidation can be performed when using on-line proteolytic digestion • ZrO_2_ and TiO_2_ may bind to phosphoproteins • Other nonphospholipid contaminants are not removed
(ZrO_2_/TiO_2_) Phospholipid trap column [62]	• Delipidation using ZrO_2_ or TiO_2_ • Can perform on-line delipidation at protein- or peptide-level • Rapid delipidation relative to robotics • Reduced ZrO_2_/TiO_2_ consumption relative to robotics	• Requires additional BSM and ‘delipidation’ valve • Requires column washing/regeneration with potential for reduced delipidation efficiency after numerous cycles • ZrO_2_ and TiO_2_ may bind to phosphoproteins • Other nonphospholipid contaminants are not removed
SEC [63]	• Minimal modifications required to existing instrumentation • Capable of removing other nonphospholipid contaminants • Can perform on-line delipidation at protein- or peptide-level • Rapid delipidation relative to robotics	• Requires additional optimisation steps (e.g., valve switch/trapping times) • Requires additional washing of SEC column between injections • Delipidation efficiency can decrease at higher lipid quantities

### The need for increased peak capacity

One limitation of HDX-MS is the need to keep LC separations short and at low temperature to prevent unwanted deuterium/hydrogen back exchange. Consequently, the extent of peptide separation is limited to the short time in which deuterium can be retained. This is problematic for the analyses of mimetic reconstituted and *in vivo* IMP samples as they exhibit high chromatographic and MS spectral complexity from the presence of other nontarget protein contaminants; making the identification and monitoring of target peptides more challenging. Thus, instrumental developments capable of increasing LC-MS peak capacity will likely play a key role in generating high-quality IMP data in the future. Here, we highlight two approaches being taken to increase peak capacity: subzero chromatography and orthogonal IM separations.

### Increasing peak capacity via subzero chromatography

Subzero temperatures permit elongated chromatography without increasing back exchange. However, typical HDX-MS systems use mobile phases unable to perform at subzero temperatures due to increased system back-pressure and freezing. The development of subzero systems for bottom-up HDX-MS has been explored [64–66]. Venable et al. evaluated the use of mobile-phase modifiers (methanol, dimethylformamide, ethylene glycol, and formamide) to drop freezing points to –30°C [64]. Wales et al. subsequently reported 1 hr long separations at –20°C with similar back exchange to 10 min separations at 0°C [65]. Fang et al. then used subzero HDX-MS on *E. coli* lysate and found that 90 min separations at –10°C generated threefold more peptide identifications than 15 min at 0°C [66], demonstrating its potential to improve analyses of complex IMP samples.

Despite its potential, practical limitations still make routine use challenging. For example, mobile-phase modifiers can reduce peptide ionisation efficiency, something Venable et al. reported [64]. Addition of mobile-phase modifiers can also alter chromatographic performance [64–66]. Furthermore, analytical columns with nonoptimal particle size and/or geometry and are often required to circumnavigate LC maximum pressure limitations [65]; only Fang et al. having used sub-2 μm particle size for –10°C separations. Therefore, further work is required to integrate ultrahigh-pressure capacity LC pumps, so that columns with ideal geometry and/or particle size can be utilised to maximise chromatographic peak capacity.

### Increasing peak capacity via orthogonal IM separations

Another strategy for increasing LC-MS peak capacity is on-line IM, which provides orthogonal peptide separation in the gas-phase [67]. This can ease spectral deconvolution of co-eluting peptides, thereby enhancing peptide identification/monitoring without elongated analyses times. This has been demonstrated in several publications utilising IM in bottom-up ‘omics’ applications [68–71].

Iacob et al. were the first to evaluate traveling wave-ion mobility (TWIM) in HDX-MS applications [72]. They observed that overlapping spectra of two co-eluting peptides prevented accurate determination of deuterium uptake. However, by applying TWIM, the respective isotopic distributions could be separated based on their IM drift times, allowing generation of processed spectra for each ion and accurate determination of their deuterium uptake. Cryar et al. also evaluated TWIM in HDX-MS by comparing results in MS^e^ and TWIM-MS^e^ modes of operation [73]. They reported that IM increased peptide output for all assessed targets, with the largest improvements observed in the most complex sample (likely due to the increased probability of co-eluting peptides). Therefore, orthogonal IM separations hold great potential to improve analyses of complex IMP samples.

More recently, Giles et al. developed cyclic ion mobility (cIM); a TWIM separator with circular geometry that permits scalable, multipass IM separations, thereby increasing available peak capacity provided in the IM dimension [74]. Here, they demonstrated that cIM resolution increases with the square root of the number of racetrack passes. Thus, by optimising cIM parameters, this device has potential to further improve available peak capacity for HDX-MS applications. This technology has been commercialised as the Waters SELECT SERIES cyclic IMS.

## Concluding remarks

The past decade has seen extensive integration of membrane mimetics into HDX-MS workflows, allowing practitioners to probe IMP dynamics within increasingly native-like, and even *in vivo*, lipid environments. Here, we report an inventory of HDX-MS work carried out on lipid-containing mimetic reconstituted and *in vivo* IMPs and highlight strategies used to improve their HDX-MS amenability.

The advantage of native-like lipid environments is their ability to provide physiologically relevant information. Thus, the field is naturally shifting towards increasingly complex native-like mimetic and *in vivo* systems, which can generate a more accurate picture of IMP mechanism of action. However, it is important to note that such systems may not always be appropriate for the desired experimental objective. For example, whilst they provide comparatively less native-like conditions, nanodiscs allow fine tuning of lipid content, as well as access of exogenous ligands, substrates, and binding partners to both sides of the IMP simultaneously. Consequently, mimetic choice should be determined on an experiment-by-experiment basis, and synergistic HDX-MS results of IMPs in several different mimetics (and its native *in vivo* environment) are likely to provide the most comprehensive characterisation.

The increasing complexity of native-like and *in vivo* systems also bring with them increased quantities of contaminants that can impair downstream LC-MS analyses. Thus, advancements in automated delipidation and LC-MS instrumentation will undoubtably pave the way to tackle the most challenging IMP targets under complex physiological conditions in the future.

Advancements in subzero chromatography and IM have been demonstrated to increase LC-MS peak capacity. As such, the continued development of instruments with these capabilities is likely to improve the analyses of complex protein mixtures derived from native-like mimetic and *in vivo* systems. Automated delipidation strategies have potential to improve sample delipidation efficiency and reduce measurement error across replicate experiments. Whilst these strategies are currently being explored independently of one another, we envision HDX-MS systems utilising multiple automated delipidation processes, in tandem, to maximise their potential. The commercialisation of automated delipidation could also result in more user-friendly instruments capable of being adopted by the wider scientific community, such as industrial researchers who wish to utilise HDX-MS for the characterisation of IMP drug targets in more complex physiological environments.

## Summary

- HDX-MS has evolved to study membrane proteins in complex, native-like environments.
- Methodological advancements have enabled HDX-MS to play key roles in the structural biology toolkit.
- Automated workflows have significantly contributed to increased data quality.
- Instrumentation innovations at the LC and MS levels will enable us to tackle challenging IMP targets in physiological conditions.

## Abbreviations

β2AR: β2-adrenergic
ABC: ATP-binding cassette
BA: bongkrekic acid
BmrA: Bacillus multidrug-resistance ATP
BSM: binary solvent manager
CATR: carboxyatractyloside
CHAPS: 3-((3-cholamidopropyl) dimethylammonio)-1-propanesulphonate
CHAPSO: 3-((3-cholamidopropyl) dimethylammonio)-2-hydroxy-1-propanesulphonate
cIM: cyclic ion mobility
CL: cardiolipin
DDM: detergent *n*-dodecyl β-D-maltoside
DMPC: 1,2-dimyristoyl-*sn*-glycero-3-phosphocholine
DOPC: 1,2-dioleoyl-*sn*-glycero-3-phosphocholine
ECL: extra cellular loop
GGCX: gamma-glutamyl carboxylase
GPCR: G protein-coupled receptor
GRK5: GPCR kinase 5
HDX-MS: hydrogen/deuterium exchange-mass spectrometry
HPLC: high-performance liquid chromatography
ICH: intracellular helice
IF: inward facing
IM: ion mobility
IMAC: immobilised metal ion affinity chromatography
IMP: integral membrane protein
IMV: inverted membrane vesicle
LacY: lactose permease
LC-MS: liquid chromatography-mass spectrometry
LeuT: leucine transporter
MD: molecular dynamics
MFS: major facilitator superfamily
MSP: membrane scaffold protein
NBD: nucleotide-binding domain
NSS: neurotransmitter:sodium symporter
OF: outward facing
OM: outer membrane
OMV: outer membrane vesicle
PC: phosphatidylcholine
PE: phosphatidylethanolamine
P-gp: P-glycoprotein
PMF: proton motive force
POPC: 1-palmitoyl-2-oleoyl-sn-glycero-3-phosphocholine
POPG: 1-hexadecanoyl-2-(9Z-octadecenoyl)-sn-glycero-3-phosphoglycerol
Sap A: saposin A
SDS: sodium dodecyl sulfate
SEC: size exclusion chromatography
SMA: styrene-maleic acid
SMALP: styrene-maleic acid lipid particle
TCA: trichloroacetic acid
TiO2: titanium dioxide
TM: transmembrane
TMD: transmembrane domain
TWIM: traveling wave-ion mobility
UPLC: ultra-performance liquid chromatography
VKD: vitamin K-dependant
WT: wild type
XyIE: D-xylose-proton symporter
ZrO2: zirconium dioxide

## References

[1] Overington J., Al-Lazikani B. and Hopkins A. (2006) How many drug targets are there? Nat. Rev. Drug Discov. 5, 993–996 10.1038/nrd2199 17139284

[2] Sriram K. and Insel P.A. (2018) G Protein-coupled receptors as targets for approved drugs: how many targets and how many drugs? Mol. Pharmacol. 93, 251–258 10.1124/mol.117.111062 29298813 PMC5820538

[3] Harayama T. and Riezman H. (2018) Understanding the diversity of membrane lipid composition. Nat. Rev. Mol. Cell Biol. 19, 281–296 10.1038/nrm.2017.138 29410529

[4] Seddon A.M., Curnow P. and Booth P.J. (2004) Membrane proteins, lipids and detergents: not just a soap opera. Biochim. Biophys. Acta 1666, 105–117 10.1016/j.bbamem.2004.04.011 15519311

[5] Thoma J. and Burmann B.M. (2020) Fake It ‘Till You Make It-the pursuit of suitable membrane mimetics for membrane protein biophysics. Int. J. Mol. Sci. 22, 10.3390/ijms22010050 33374526 PMC7793082

[6] Moller I.R., Merkle P.S., Calugareanu D., Comamala G., Schmidt S.G., Loland C.J. et al. (2020) Probing the conformational impact of detergents on the integral membrane protein LeuT by global HDX-MS. J. Proteomics 225, 103845 10.1016/j.jprot.2020.103845 32480080

[7] Majeed S., Ahmad A.B., Sehar U. and Georgieva E.R. (2021) Lipid membrane mimetics in functional and structural studies of integral membrane proteins. Membranes (Basel) 11, 685, 10.3390/membranes11090685 34564502 PMC8470526

[8] Miller M.D. and Phillips G.N. Jr (2021) Moving beyond static snapshots: protein dynamics and the Protein Data Bank. J. Biol. Chem. 296, 100749 10.1016/j.jbc.2021.100749 33961840 PMC8164045

[9] Weis D.D. (2016) Hydrogen Exchange Mass Spectrometry of Proteins: Fundamentals, Methods, and Applications, John Wiley & Sons, Chichester, UK

[10] Hodge E.A., Benhaim M.A. and Lee K.K. (2020) Bridging protein structure, dynamics, and function using hydrogen/deuterium-exchange mass spectrometry. Protein Sci. 29, 843–855 10.1002/pro.3790 31721348 PMC7096709

[11] Engen J.R., Botzanowski T., Peterle D., Georgescauld F. and Wales T.E. (2021) Developments in hydrogen/deuterium exchange mass spectrometry. Anal. Chem. 93, 567–582 10.1021/acs.analchem.0c04281 33112590

[12] James E.I., Murphree T.A., Vorauer C., Engen J.R. and Guttman M. (2022) Advances in hydrogen/deuterium exchange mass spectrometry and the pursuit of challenging biological systems. Chem. Rev. 122, 7562–7623 10.1021/acs.chemrev.1c00279 34493042 PMC9053315

[13] Pyle E., Kalli A.C., Amillis S., Hall Z., Lau A.M., Hanyaloglu A.C. et al. (2018) Structural lipids enable the formation of functional oligomers of the eukaryotic purine symporter UapA. Cell Chem. Biol. 25, 840e4–848e4 10.1016/j.chembiol.2018.03.011 29681524 PMC6058078

[14] Bolla J.R., Agasid M.T., Mehmood S. and Robinson C.V. (2019) Membrane protein-lipid interactions probed using mass spectrometry. Annu. Rev. Biochem. 88, 85–111 10.1146/annurev-biochem-013118-111508 30901263

[15] Nji E., Chatzikyriakidou Y., Landreh M. and Drew D. (2018) An engineered thermal-shift screen reveals specific lipid preferences of eukaryotic and prokaryotic membrane proteins. Nat. Commun. 9, 4253 10.1038/s41467-018-06702-3 30315156 PMC6185904

[16] Martens C., Shekhar M., Borysik A.J., Lau A.M., Reading E., Tajkhorshid E. et al. (2018) Direct protein-lipid interactions shape the conformational landscape of secondary transporters. Nat. Commun. 9, 4151 10.1038/s41467-018-06704-1 30297844 PMC6175955

[17] Sligar S.G. and Denisov I.G. (2021) Nanodiscs: a toolkit for membrane protein science. Protein Sci. 30, 297–315 10.1002/pro.3994 33165998 PMC7784751

[18] Denisov I.G. and Sligar S.G. (2017) Nanodiscs in membrane biochemistry and biophysics. Chem. Rev. 117, 4669–4713 10.1021/acs.chemrev.6b00690 28177242 PMC5805400

[19] Bayburt T.H. and Sligar S.G. (2010) Membrane protein assembly into nanodiscs. FEBS Lett. 584, 1721–1727 10.1016/j.febslet.2009.10.024 19836392 PMC4758813

[20] Redhair M., Clouser A.F. and Atkins W.M. (2019) Hydrogen-deuterium exchange mass spectrometry of membrane proteins in lipid nanodiscs. Chem. Phys. Lipids. 220, 14–22 10.1016/j.chemphyslip.2019.02.007 30802434 PMC6480397

[21] Martens C. and Politis A. (2020) A glimpse into the molecular mechanism of integral membrane proteins through hydrogen-deuterium exchange mass spectrometry. Protein Sci. 29, 1285–1301 10.1002/pro.3853 32170968 PMC7255514

[22] Hebling C.M., Morgan C.R., Stafford D.W., Jorgenson J.W., Rand K.D. and Engen J.R. (2010) Conformational analysis of membrane proteins in phospholipid bilayer nanodiscs by hydrogen exchange mass spectrometry. Anal. Chem. 82, 5415–5419 10.1021/ac100962c 20518534 PMC2895417

[23] Parker C.H., Morgan C.R., Rand K.D., Engen J.R., Jorgenson J.W. and Stafford D.W. (2014) A conformational investigation of propeptide binding to the integral membrane protein gamma-glutamyl carboxylase using nanodisc hydrogen exchange mass spectrometry. Biochemistry 53, 1511–1520 10.1021/bi401536m 24512177 PMC3970815

[24] Adhikary S., Deredge D.J., Nagarajan A., Forrest L.R., Wintrode P.L. and Singh S.K. (2017) Conformational dynamics of a neurotransmitter:sodium symporter in a lipid bilayer. Proc. Natl. Acad. Sci. U.S.A. 114, E1786–E1795 10.1073/pnas.1613293114 28223522 PMC5347597

[25] Locher K.P. (2016) Mechanistic diversity in ATP-binding cassette (ABC) transporters. Nat. Struct. Mol. Biol. 23, 487–493 10.1038/nsmb.3216 27273632

[26] Bosshart P.D. and Fotiadis D. (2019) Secondary active transporters. Subcell. Biochem. 92, 275–299 10.1007/978-3-030-18768-2_9 31214990

[27] Kazmier K., Sharma S., Quick M., Islam S.M., Roux B., Weinstein H. et al. (2014) Conformational dynamics of ligand-dependent alternating access in LeuT. Nat. Struct. Mol. Biol. 21, 472–479 10.1038/nsmb.2816 24747939 PMC4050370

[28] Merkle P.S., Gotfryd K., Cuendet M.A., Leth-Espensen K.Z., Gether U., Loland C.J. et al. (2018) Substrate-modulated unwinding of transmembrane helices in the NSS transporter LeuT. Sci. Adv. 4, eaar6179 10.1126/sciadv.aar6179 29756037 PMC5947982

[29] Martens C., Shekhar M., Lau A.M., Tajkhorshid E. and Politis A. (2019) Integrating hydrogen-deuterium exchange mass spectrometry with molecular dynamics simulations to probe lipid-modulated conformational changes in membrane proteins. Nat. Protoc. 14, 3183–3204 10.1038/s41596-019-0219-6 31605097 PMC7058097

[30] Li M.J., Guttman M. and Atkins W.M. (2018) Conformational dynamics of P-glycoprotein in lipid nanodiscs and detergent micelles reveal complex motions on a wide time scale. J. Biol. Chem. 293, 6297–6307 10.1074/jbc.RA118.002190 29511086 PMC5925813

[31] Clouser A.F., Alam Y.H. and Atkins W.M. (2021) Cholesterol asymmetrically modulates the conformational ensemble of the nucleotide-binding domains of P-glycoprotein in lipid nanodiscs. Biochemistry 60, 85–94 10.1021/acs.biochem.0c00824 33350827

[32] Clouser A.F. and Atkins W.M. (2022) Long range communication between the drug-binding sites and nucleotide binding domains of the efflux transporter ABCB1. Biochemistry 61, 730–740 10.1021/acs.biochem.2c00056 35384651 PMC9022228

[33] Chaptal V., Zampieri V., Wiseman B., Orelle C., Martin J., Nguyen K.-A. et al. (2022) Substrate-bound and substrate-free outward-facing structures of a multidrug ABC exporter. Sci. Adv. 8, eabg9215 10.1126/sciadv.abg9215 35080979 PMC8791611

[34] Zampieri V., Hilpert C., Garnier M., Gestin Y., Delolme S., Martin J. et al. (2021) The Det.Belt Server: a tool to visualize and estimate amphipathic solvent belts around membrane proteins. Membranes (Basel) 11, 459 10.3390/membranes11070459 34206634 PMC8307592

[35] Frauenfeld J., Loving R., Armache J.P., Sonnen A.F., Guettou F., Moberg P. et al. (2016) A saposin-lipoprotein nanoparticle system for membrane proteins. Nat. Methods 13, 345–351 10.1038/nmeth.3801 26950744 PMC4894539

[36] Lloris-Garcerá P., Klinter S., Chen L., Skynner M.J., Löving R. and Frauenfeld J. (2020) DirectMX - one-step reconstitution of membrane proteins from crude cell membranes into salipro nanoparticles. Front. Bioeng. Biotechnol. 8, 215, 10.3389/fbioe.2020.00215 32266242 PMC7096351

[37] Flayhan A., Mertens H.D.T., Ural-Blimke Y., Martinez Molledo M., Svergun D.I. and Low C. (2018) Saposin lipid nanoparticles: a highly versatile and modular tool for membrane protein research. Structure 26, 345e5–355e5 10.1016/j.str.2018.01.007 29413323 PMC5807053

[38] Zhou F., Yang Y., Chemuru S., Cui W., Liu S., Gross M. et al. (2021) Footprinting mass spectrometry of membrane proteins: ferroportin reconstituted in saposin A picodiscs. Anal. Chem. 93, 11370–11378 10.1021/acs.analchem.1c02325 34383472 PMC8903032

[39] Stroud Z., Hall S.C.L. and Dafforn T.R. (2018) Purification of membrane proteins free from conventional detergents: SMA, new polymers, new opportunities and new insights. Methods 147, 106–117 10.1016/j.ymeth.2018.03.011 29608964

[40] Ravula T., Hardin N.Z. and Ramamoorthy A. (2019) Polymer nanodiscs: advantages and limitations. Chem. Phys. Lipids. 219, 45–49 10.1016/j.chemphyslip.2019.01.010 30707909 PMC6497063

[41] Fiori M.C., Zheng W., Kamilar E., Simiyu G., Altenberg G.A. and Liang H. (2020) Extraction and reconstitution of membrane proteins into lipid nanodiscs encased by zwitterionic styrene-maleic amide copolymers. Sci. Rep. 10, 9940 10.1038/s41598-020-66852-7 32555261 PMC7303149

[42] Reading E., Hall Z., Martens C., Haghighi T., Findlay H., Ahdash Z. et al. (2017) Interrogating membrane protein conformational dynamics within native lipid compositions. Angew. Chem., Int. Ed. Engl. 56, 15654–15657 10.1002/anie.201709657 29049865

[43] Sanders C.R., Hare B.J., Howard K.P. and Prestegard J.H. (1994) Magnetically-oriented phospholipid micelles as a tool for the study of membrane-associated molecules. Prog. Nucl. Magn. Reson. Spectrosc. 26, 421–444 10.1016/0079-6565(94)80012-X

[44] Diller A., Loudet C., Aussenac F., Raffard G., Fournier S., Laguerre M. et al. (2009) Bicelles: a natural ‘molecular goniometer’ for structural, dynamical and topological studies of molecules in membranes. Biochimie 91, 744–751 10.1016/j.biochi.2009.02.003 19248817 PMC2899883

[45] Khanal A., Pan Y., Brown L.S. and Konermann L. (2012) Pulsed hydrogen/deuterium exchange mass spectrometry for time-resolved membrane protein folding studies. J. Mass Spectrom. 47, 1620–1626 10.1002/jms.3127 23280751

[46] Pan Y., Brown L. and Konermann L. (2011) Hydrogen/deuterium exchange mass spectrometry and optical spectroscopy as complementary tools for studying the structure and dynamics of a membrane protein. Int. J. Mass Spectrom. 302, 3–11 10.1016/j.ijms.2010.04.011

[47] Duc N.M., Du Y., Zhang C., Lee S.Y., Thorsen T.S., Kobilka B.K. et al. (2015) Effective application of bicelles for conformational analysis of G protein-coupled receptors by hydrogen/deuterium exchange mass spectrometry. J. Am. Soc. Mass. Spectrom. 26, 808–817 10.1007/s13361-015-1083-4 25740347 PMC4727453

[48] Komolov K.E., Du Y., Duc N.M., Betz R.M., Rodrigues J., Leib R.D. et al. (2017) Structural and functional analysis of a beta2-adrenergic receptor complex with GRK5. Cell 169, 407e16–421e16 10.1016/j.cell.2017.03.047 28431242 PMC5526774

[49] Rigaud J.L.L.D. (2003) Reconstitution of membrane proteins into liposomes. Methods Enzymol. 372, 65–86 10.1016/S0076-6879(03)72004-7 14610807

[50] Yao X., Fan X. and Yan N. (2020) Cryo-EM analysis of a membrane protein embedded in the liposome. Proc. Natl. Acad. Sci. U.S.A. 117, 18497–18503 10.1073/pnas.2009385117 32680969 PMC7414195

[51] Demmers J.A., van Duijn E., Haverkamp J., Greathouse D.V., Koeppe R.E. 2nd, Heck A.J. et al. (2001) Interfacial positioning and stability of transmembrane peptides in lipid bilayers studied by combining hydrogen/deuterium exchange and mass spectrometry. J. Biol. Chem. 276, 34501–34508 10.1074/jbc.M101401200 11435420

[52] Demmers J.A.A., Haverkamp J., Heck A.J.R., Koeppe R.E. and Killian J.A. (2000) Electrospray ionization mass spectrometry as a tool to analyze hydrogen/deuterium exchange kinetics of transmembrane peptides in lipid bilayers. Proc. Natl. Acad. Sci. U.S.A. 97, 3189–3194 10.1073/pnas.97.7.3189 10725361 PMC16214

[53] Hansen R.K., Broadhurst R.W., Skelton P.C. and Arkin I.T. (2002) Hydrogen/deuterium exchange of hydrophobic peptides in model membranes by electrospray ionization mass spectrometry. J. Am. Soc. Mass Spectrom. 13, 1376–1387 10.1016/S1044-0305(02)00702-X 12484457

[54] Anderson K.W., Gallagher E.S. and Hudgens J.W. (2018) Automated removal of phospholipids from membrane proteins for H/D exchange mass spectrometry workflows. Anal. Chem. 90, 6409–6412 10.1021/acs.analchem.8b00429 29723469 PMC6050989

[55] Rey M., Forest E. and Pelosi L. (2012) Exploring the conformational dynamics of the bovine ADP/ATP carrier in mitochondria. Biochemistry 51, 9727–9735 10.1021/bi300759x 23136955

[56] Rey M., Man P., Clemencon B., Trezeguet V., Brandolin G., Forest E. et al. (2010) Conformational dynamics of the bovine mitochondrial ADP/ATP carrier isoform 1 revealed by hydrogen/deuterium exchange coupled to mass spectrometry. J. Biol. Chem. 285, 34981–34990 10.1074/jbc.M110.146209 20805227 PMC2966112

[57] Mehmood S., Domene C., Forest E. and Jault J.M. (2012) Dynamics of a bacterial multidrug ABC transporter in the inward- and outward-facing conformations. Proc. Natl. Acad. Sci. U.S.A. 109, 10832–10836 10.1073/pnas.1204067109 22711831 PMC3390859

[58] Vahidi S., Bi Y., Dunn S.D. and Konermann L. (2016) Load-dependent destabilization of the gamma-rotor shaft in FOF1 ATP synthase revealed by hydrogen/deuterium-exchange mass spectrometry. Proc. Natl. Acad. Sci. U.S.A. 113, 2412–2417 10.1073/pnas.1520464113 26884184 PMC4780623

[59] Donnarumma D., Maestri C., Giammarinaro P.I., Capriotti L., Bartolini E., Veggi D. et al. (2018) Native state organization of outer membrane porins unraveled by HDx-MS. J. Proteome Res. 17, 1794–1800 10.1021/acs.jproteome.7b00830 29619829

[60] Zmyslowski A.M., Baxa M.C., Gagnon I.A. and Sosnick T.R. (2022) HDX-MS performed on BtuB in E. coli outer membranes delineates the luminal domain's allostery and unfolding upon B12 and TonB binding. Proc. Natl. Acad. Sci. U.S.A. 119, e2119436119 10.1073/pnas.2119436119 35549554 PMC9171809

[61] Lin X., Zmyslowski A.M., Gagnon I.A., Nakamoto R.K. and Sosnick T.R. (2022) Development of in vivo HDX-MS with applications to a TonB-dependent transporter and other proteins. Protein Sci. 31, e4402 10.1002/pro.4402 36040258 PMC9382693

[62] Hammerschmid D.C.V., Bailey C., Russell Lewis B., Politis A., Morris M. et al. (2022) Chromatographic phospholipid trapping for automated H/D exchange mass spectrometry analysis of membrane protein-lipid assemblies. Chem. Rxiv Cambridge: Cambridge Open Engage (This content is a preprint and has not been peer-reviewed) 10.1021/acs.analchem.2c04876 PMC9909672 36706021

[63] Calvaresi V., Redsted A., Norais N. and Rand K.D. (2021) Hydrogen-deuterium exchange mass spectrometry with integrated size-exclusion chromatography for analysis of complex protein samples. Anal. Chem. 93, 11406–11414 10.1021/acs.analchem.1c01171 34387074

[64] Venable J.D., Okach L., Agarwalla S. and Brock A. (2012) Subzero temperature chromatography for reduced back-exchange and improved dynamic range in amide hydrogen/deuterium exchange mass spectrometry. Anal. Chem. 84, 9601–9608 10.1021/ac302488h 23025328 PMC3494095

[65] Wales T.E., Fadgen K.E., Eggertson M.J. and Engen J.R. (2017) Subzero Celsius separations in three-zone temperature controlled hydrogen deuterium exchange mass spectrometry. J. Chromatogr. A. 1523, 275–282 10.1016/j.chroma.2017.05.067 28596009 PMC5675777

[66] Fang M., Wang Z., Cupp-Sutton K.A., Welborn T., Smith K. and Wu S. (2021) High-throughput hydrogen deuterium exchange mass spectrometry (HDX-MS) coupled with subzero-temperature ultrahigh pressure liquid chromatography (UPLC) separation for complex sample analysis. Anal. Chim. Acta 1143, 65–72 10.1016/j.aca.2020.11.022 33384131 PMC8265693

[67] Dodds J.N. and Baker E.S. (2019) Ion mobility spectrometry: fundamental concepts, instrumentation, applications, and the road ahead. J. Am. Soc. Mass. Spectrom. 30, 2185–2195 10.1007/s13361-019-02288-2 31493234 PMC6832852

[68] Arthur K.L., Turner M.A., Reynolds J.C. and Creaser C.S. (2017) Increasing peak capacity in nontargeted omics applications by combining full scan field asymmetric waveform ion mobility spectrometry with liquid chromatography-mass spectrometry. Anal. Chem. 89, 3452–3459 10.1021/acs.analchem.6b04315 28230966

[69] Causon T.J. and Hann S. (2015) Theoretical evaluation of peak capacity improvements by use of liquid chromatography combined with drift tube ion mobility-mass spectrometry. J. Chromatogr. A. 1416, 47–56 10.1016/j.chroma.2015.09.009 26372446

[70] Canterbury J.D., Yi X., Hoopmann M.R. and MacCoss M.J. (2008) Assessing the dynamic range and peak capacity of nanoflow LC-FAIMS-MS on an ion trap mass spectrometer for proteomics. Anal. Chem. 80, 6888–6897 10.1021/ac8004988 18693747 PMC2818878

[71] Haynes S.E., Polasky D.A., Dixit S.M., Majmudar J.D., Neeson K., Ruotolo B.T. et al. (2017) Variable-velocity traveling-wave ion mobility separation enhancing peak capacity for data-independent acquisition proteomics. Anal. Chem. 89, 5669–5672 10.1021/acs.analchem.7b00112 28471653 PMC5623091

[72] Iacob R.E., Murphy J.P. 3rd and Engen J.R. (2008) Ion mobility adds an additional dimension to mass spectrometric analysis of solution-phase hydrogen/deuterium exchange. Rapid Commun. Mass Spectrom. 22, 2898–2904 10.1002/rcm.3688 18727141 PMC9335573

[73] Cryar A., Groves K. and Quaglia M. (2017) Online hydrogen-deuterium exchange traveling wave ion mobility mass spectrometry (HDX-IM-MS): a systematic evaluation. J. Am. Soc. Mass. Spectrom. 28, 1192–1202 10.1007/s13361-017-1633-z 28374315 PMC5438439

[74] Giles K., Ujma J., Wildgoose J., Pringle S., Richardson K., Langridge D. et al. (2019) A cyclic ion mobility-mass spectrometry system. Anal. Chem. 91, 8564–8573 10.1021/acs.analchem.9b01838 31141659

[75] Lau A.M., Claesen J., Hansen K. and Politis A. (2021) Deuteros 2.0: peptide-level significance testing of data from hydrogen deuterium exchange mass spectrometry. Bioinformatics 37, 270–272 10.1093/bioinformatics/btaa677 32722756 PMC8055227

